# Structure–Property Correlation in Natural Rubber Nanocomposite Foams: A Comparison between Nanoclay and Cellulose Nanofiber Used as Nanofillers

**DOI:** 10.3390/polym15214223

**Published:** 2023-10-25

**Authors:** Bunsita Wongvasana, Bencha Thongnuanchan, Abdulhakim Masa, Hiromu Saito, Tadamoto Sakai, Natinee Lopattananon

**Affiliations:** 1 Department of Rubber Technology and Polymer Science, Faculty of Science and Technology, Prince of Songkla University, Pattani 94000, Thailand; wongvasana.b@gmail.com (B.W.); bencha.t@psu.ac.th (B.T.); 2 Rubber Engineering & Technology Program, International College, Prince of Songkla University, Songkhla 90110, Thailand; abdulhakim.m@psu.ac.th; 3 Department of Organic and Polymer Materials Chemistry, Tokyo University of Agriculture and Technology, Koganei-shi 184-8588, Tokyo, Japan; 4 Organization for Innovation & Social Collaboration, Shizuoka University, 3-5-1 Johoku, Naka-ku, Hamamatsu City 432-8011, Shizuoka, Japan; sakai.tadamoto@shizuoka.ac.jp

**Keywords:** natural rubber, nanocomposite foams, nanoclay, cellulose nanofibers, reinforcement behavior

## Abstract

Nanocomposite foams of natural rubber (NR) with 5 phr of two kinds of nanofillers, nanoclay (NC) and cellulose nanofiber (CNF), were produced using the latex mixing method and foaming with azodicarbonamide. The effect of the nanofiller on the structure and mechanical properties of NR foams was investigated through SEM, TEM, tensile tests, WAXD, and compression set measurements. Smaller cells with a narrower distribution were attained in the NC/NR foam when compared to the NR and CNF/NR foams, and the expansion ratio was larger due to the suppression of the shrinkage in the NC/NR foam. The foaming of the NR nanocomposites reduced the size of the filler aggregates and improved the dispersion and alignment of nanofillers in the cell walls. The addition of NC and CNF enhanced the tensile strength of the NR foam by 139% and 62%, respectively, without sacrificing the excellent strain of the NR, due to the acceleration of the strain-induced crystallization and small size of the filler aggregates. The compression set of the NR foam could also be reduced in the NC/NR foam compared with the NR and CNF/NR foams.

## 1. Introduction

Currently, rubbery foams are one of the most versatile materials. They have been widely used in the areas of construction, transportation, agriculture, furniture and bedding, medical applications, and packaging due to their superior properties to their solid counterparts in terms of light weight; excellent insulating abilities (sound and heat); good energy absorption (shock, impact, and vibration); buoyancy; and low cost [1,2,3,4,5].

Natural rubber (NR) is a commonly used commercial biopolymeric material for manufacturing automotive tires, gloves, gaskets, seals, and so on. Overall, NR shows high tensile strength, high resistance to tear, good resilience, good elastomeric recovery, and low heat build-up [6,7], which make it very attractive for the preparation of rubber foams in both scientific research and industrial developments. In the past two decades, several authors successfully prepared foam materials based on NR, and they showed that the properties of the NR foams were influenced by their cellular structure, which in turn depended on processing factors such as the foaming temperature, time, pressure, and preparation method [8,9,10,11,12,13,14]. In addition, the NR grade, crosslink density, blowing agent, and filler were the key contributors to the preparation and properties of the NR foams [2,10,11,15,16]. Although foams made of NR have been developed, it has been found that NR foams still exhibit poor cellular structure (i.e., large cells with a broad cell size distribution) and relatively unfavorable mechanical properties.

It has been reported that the cell nucleation and mechanical properties of NR foams are improved by using nanofiller [8,9,13,14,17]. For instance, Kim et al. [8] investigated the effect of carbon black on the morphology and mechanical properties of NR foams. The addition of carbon black improved the cellular structure of NR foams by reducing the cell size and cell size distribution, which enhanced the mechanical properties of NR nanocomposite foams. Similarly, Vahidifar et al. [13] characterized the foaming behavior and mechanical properties of NR by using carbon black as a filler and azodicarbonamide (ADC) as a blowing agent. The incorporation of carbon black (0–20 phr) into NR resulted in a smaller cell size and higher cell density, depending on the amount of carbon black. The tensile modulus and strength of carbon-black-filled NR foams were increased upon the incorporation of carbon black. The enhancement of the mechanical properties was attributed to the improvement of the cellular structure. Recently, nanoclay and graphene were used to improve the properties of NR foams [14,17]. By the addition of these nanofillers, the mechanical properties of NR nanocomposite foams were significantly improved due to the reinforcing effect of the nanofillers and the improved cellular structure. Currently, the dispersion of nanofillers from natural resources to foam polymers is gaining popularity among researchers and industries. As for natural nanofillers, nanoclay (NC), derived from naturally occurring clay minerals, and cellulose nanofiber (CNF), originating from plant-based cellulose, are of great interest because they provide not only mechanical property enhancement, but also a solution to environmental concerns regarding production, utilization, and waste. Although the importance of NC and CNF as cell nucleating agents and reinforcements for many polymer foams has been addressed [18,19,20,21,22,23,24,25,26,27,28,29,30,31,32,33], details of the effects of NC and CNF on the structure and mechanical performance of NR foams have not been clarified.

The excellent mechanical properties of NR are closely related to its significant strain-induced crystallization. Recently, Wongvasana and co-workers [34] studied and compared the structure–property relationship of NR nanocomposites reinforced with NC and CNF at a filling amount of 5 phr. They showed that the addition of NC and CNF improved the mechanical properties and accelerated the strain-induced crystallization of NR. They also proposed that the acceleration of the strain-induced crystallization behaviors of NR nanocomposites in the presence of NC and CNF dispersion should be considered a reinforcing mechanism responsible for the increased tensile properties of NC/NR and CNF/NR nanocomposites. However, in the case of NR nanocomposite foams, the strain-induced crystallization behavior may be different from that of unfoamed solid NR nanocomposites due to the dispersed gas bubbles within the NR nanocomposite foams. Obviously, the studies in the past did not investigate and clarify the role of nanofillers and cellular structure in the mechanism for strengthening NR-based foams reinforced with NC and CNF, resulting in difficulties controlling the mechanical properties of NR foam products.

Therefore, this article is devoted to studying the effects of nanofillers on the foaming behavior and reinforcement mechanism of rubber foams based on NC/NR and CNF/NR nanocomposites. Pristine NC and CNFs were used to reinforce the NR foams because they are inexpensive and present no health and environmental risks related to the handling of chemicals and wastes. The structure and properties of two different kinds of NR nanocomposite foams were compared. The NR was mixed with NC and CNF at a fixed concentration (5 phr) and foamed using azodicarbonamide (ADC) as a foaming agent. NR foam without nanofiller was also prepared for comparison. The foaming process and mechanical properties of these two NR nanocomposite foams were studied by SEM, TEM, tensile tests, and compression set tests. The strain-induced crystallization of NR nanocomposite foams reinforced with NC and CNF during tensile deformation was monitored through WAXD measurement.

## 2. Materials and Methods

### 2.1. Materials

High-ammonia (HA) concentrated natural rubber (NR) latex with a dry rubber content (DRC) of 60%, density of 0.92 g/cm^3^, viscosity of 80 mPa.s, and pH of 10.5 was supplied by Yala Latex Co., Ltd. (Yala, Thailand). Sodium montmorillonite (clay, Kunipia-F^®^) was kindly provided by Kunimine Industries Co., Ltd. (Tokyo, Japan). Cellulose nanofiber (CNF, Nanoforest-S) made from wood pulp using the aqueous counter-collision (ACC) method was kindly supplied by Chuetsu Pulp and Paper Co., Ltd. (Tokyo, Japan). Azodicarbonamide (ADC) was provided as a foaming agent. Dicumyl peroxide (DCP) was supplied as a crosslinking agent. Zinc oxide (ZnO) and stearic acid were used as kickers to decrease the decomposition temperature of the blowing agent in the foaming process. 2,2,4-trimethyl-1,2-dihydroquinone (TMQ) was used as an antioxidant. Paraffinic oil was used as a plasticizer to reduce the viscosity of the NR. The grade and supplier of the rubber, fillers, and additives are listed in Table 1.

### 2.2. Preparation of NC/NR and CNF/NR Nanocomposite Foams

**Figure 1:**
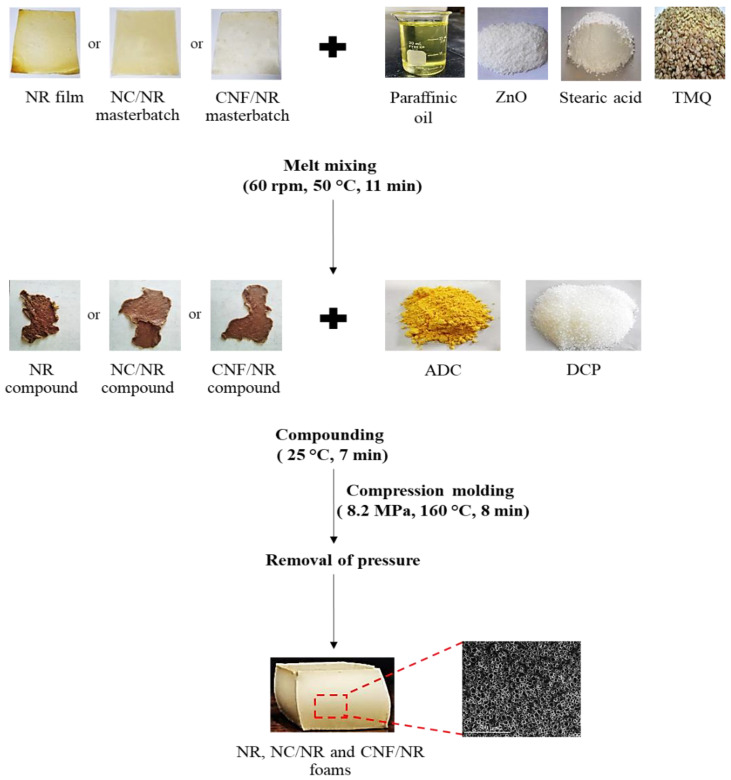
Schematic diagram of preparation of NR and NR nanocomposite foams.

The fabrication of NR nanocomposite foams was carried out by the systematic process shown in Figure 1. Two different kinds of masterbatches of NR nanocomposites were prepared via the latex mixing method, as outlined in literature [34], and the amount of NC or CNF was fixed at 5 phr. NR without nanofiller was also prepared via the same method and used as a reference. The dried NR and masterbatches of NR nanocomposites were melt-mixed with ZnO, stearic acid, paraffinic oil, and TMQ in an internal mixer (CT internal mixer) (Chareon TUT Co., Ltd., Samutprakan, Thailand) at a temperature of 50 °C with a rotor speed of 60 rpm for 11 min. The obtained NR and NR nanocomposite compounds were mixed with ADC and DCP in a two-roll mill at room temperature for 7 min. The formulations of the NR and NR nanocomposite compounds are given in Table 2. After that, the compounds were hot-pressed in a mold under a pressure of 8.2 MPa and temperature of 160 °C for the crosslinking of the NR. Finally, the pressure was removed, and the samples were allowed to expand for foaming.

### 2.3. Characterizations

#### 2.3.1. Transmission Electron Microscopy (TEM)

TEM analysis was used to investigate the dispersion of NC and CNF in the solid and foamed NR nanocomposites. An ultra-thin section (ca. 100 nm) of the sample was cut with a diamond knife at −100 °C using RMC MT-XL, RMC Products Group (Ventana Medical System, Inc., Oro Valley, AZ, USA). The thin sections of the samples were observed using a JEOL JEM 2010 (JEOL Co., Tokyo, Japan) at an accelerating voltage of 200 kV and a magnification of ×2500 to ×4000.

#### 2.3.2. Density Measurement

Density was determined by water displacement using an electronic densimeter, model MD-300S (Alfa Mirage Co., Ltd., Tokyo, Japan), according to ASTM D3575 [35]. This method was applied to both the solid and foamed samples.

The volume expansion ratio of the foam (Ø) was determined using Equation (1).

(1) $$\emptyset =\frac{\rho }{\rho_{f}}$$

where *ρ* and *ρ_f_* are the densities of the samples before and after foaming, respectively.

#### 2.3.3. Scanning Electron Microscopy (SEM)

SEM analysis was used to observe the cellular structure of the NR and nanocomposite foams. The sample pieces of NR and NR nanocomposite foams were cut with a razor into thin slices. The razor-cut samples were scanned under a scanning electron microscope (FEI, Quanta 400, Eindhoven, The Netherlands) using low-vacuum mode. The SEM images of the razor-cut surface were taken at ×100 magnification. The cell size, cell size distribution, and the 3D cell density (number of bubbles per unit volume) were evaluated from the SEM images with the aid of a software program (Image J 1.8.0). In an isotropic polymeric foam, the cell density is estimated by assuming that the foam is isotropic with a uniform distribution of spherical bubbles in all directions, according to Equation (2).

(2) $$3D\;\mathrm{cell}\;\mathrm{density}={\left(\frac{nM^{2}}{A}\right)}^{\frac{3}{2}}\times \emptyset$$

#### 2.3.4. Shrinkage Measurement

Shrinkage was used to estimate dimensional stability after the foaming of the NR and nanocomposite foams. The shrinkage of the foam was measured by calculating the changes in the volume of the NR foam both before and after the stabilization of the foam. The shrinkage was calculated as follows:

(3) $$\mathrm{Shrinkage}\;\left(\%\right)\;=\left[\frac{VE_{i}-VE_{f}}{VE_{i}}\right]\times \emptyset$$

where *VE_i_* and *VE_f_* are the volume expansions of the samples before and after foaming, respectively.

#### 2.3.5. Wide-Angle X-ray Diffraction (WAXD) Measurement

To estimate the degree of crystallinity in the NR and NR nanocomposite foams during tensile stretching, wide angle X-ray diffraction (WAXD) measurement was performed using a NANO-Viewer system (Rigaku Co., Ltd., Tokyo, Japan) and an imaging plate (IP) two-dimensional detector (Fujifilm BAS-SR 127, Fujifilm Corp., Tokyo, Japan), as described detail in the literature [34]. The sample was stretched in stages after WAXD measurement at a fixed strain using a miniature tensile machine (Imoto Machinery Co., Ltd., Kyoto, Japan). The exposure time was 15 min at room temperature (20 °C). The scattering intensity was corrected with respect to the exposure time, the sample thickness, and the transmittance.

The area of the crystalline diffraction peaks and that of the amorphous halo were obtained by the curve fitting of the peaks using Origin^®^9.1 software. The *X_c_* was calculated using Equation (4).

(4) $$X_{c}=\frac{A_{c}}{A_{c}+A_{a}}\times 100\%$$

where *A_c_* corresponds to the area of the crystalline region and *A_a_* corresponds to the area of the amorphous region.

#### 2.3.6. Mechanical Property Measurement

##### Tensile Properties

The tensile properties were measured on a Hounsfield Tensometer (H10KS, Hounsfield Test Equipment Co., Ltd., Redhill, Surrey, UK) at a temperature of 25 ± 2 °C with an extension rate of 500 ± 50 mm/min according to ASTM D3575-00. Dumbbell-shaped specimens were cut from the rubber foams. An average of ten specimens were considered for the tensile test.

##### Compression Set

The compression set under constant deflection was assessed in accordance with ASTM D1056-00 [36]. The NR and NR nanocomposite foam samples with dimensions of 2 cm × 2 cm were compressed to 50% of their original sample thickness for 22 h at 23 ± 2 °C. The load was released at the end of 22 h, and the final sample thickness was measured after 24 h at room temperature. The compression set was calculated as follows:

(5) $$\mathrm{Compression}\;\mathrm{set}=\left[\frac{\left(t_{0}-t_{1}\right)}{\left(t_{0}-t_{s}\right)}\right]\times 100\%$$

where *t*_0_ is the original thickness of the test specimen (mm), *t*_1_ is the final thickness of the test specimen (mm), and *t_s_* is the spacer thickness.

## 3. Results and Discussion

### 3.1. Cellular Structure and Foam Properties

**Figure 2:**
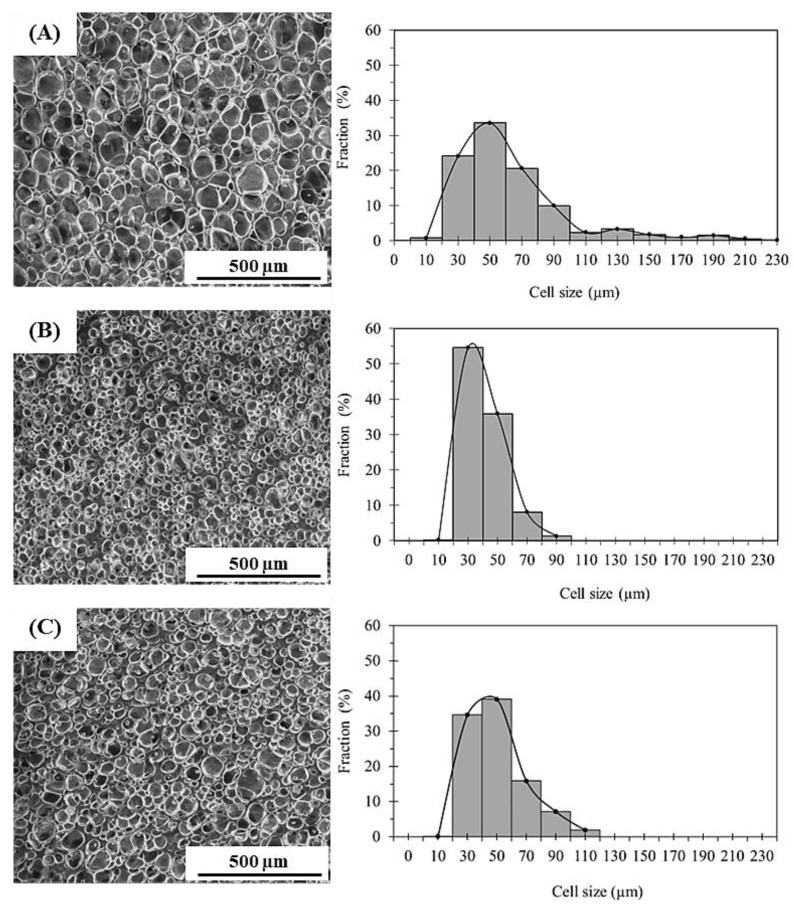
SEM images and cell size distribution of (**A**) NR, (**B**) NC/NR, and (**C**) CNF/NR foams.

Figure 2 shows SEM images and histograms of the cell size distribution for the NR and NR nanocomposite foams. The NR foam without nanofiller contained many large cells with a fairly irregular shape, displaying a broad cell size distribution ranging from 15 to 153 µm (Figure 2A). By incorporating the nanofillers, the NR foams achieved smaller cells with a narrower cell size distribution, and the cells became more spherical in shape (Figure 2B,C). The cell size distribution of the NC/NR and CNF/NR foams was in the range of about 4–87 µm and 11–108 µm, respectively.

The cell parameters (average cell size, cell density, and cell size distribution) of the NR and NR nanocomposite foams obtained from Figure 2 are tabulated in Table 3. All the NR foams exhibited closed cells with different cell size distributions. The cell size in the NR foam was about 60 µm, and the cell density (the number density of the cells) was approximately 10^5^ cells/cm^3^. By incorporating NC and CNF into the NR, the cell densities of the NR foams were greatly increased by factors of 100 and 10, respectively, and the cell sizes were markedly decreased by a factor of about 2 and 1.5, respectively. Smaller cells with a narrower distribution of cell size could be obtained in the NC/NR foam compared with the CNF/NR foam, and the cell density of the NC/NR foam was higher than that of the CNF/NR foam, i.e., the average cell sizes in the NC/NR and CNF/NR foams were about 29 µm and 42 µm, respectively, and the cell densities of the NC/NR and CNF/NR foams were about 2.4 × 10^7^ cells/cm^3^ and 3.7 × 10^6^ cells/cm^3^, respectively. For the NR nanocomposite foams obtained in this study, the cell size of less than 40 µm was smaller and the cell density of 10^6^–10^7^ cells/cm^3^ was higher than that of NR-based foams generally prepared using a chemical blowing agent and a similar amount of nanofiller, i.e., the average cell size is larger than 100 µm and the cell density is 10^2^–10^4^ cells/cm^3^ [13,14,17]. Thus, it is suggested that the nanofillers worked as a bubble nucleating agent for the formation of finer cellular structures in the NR foams, and the nucleating agent effect of NC was stronger than that of CNF.

Cotton and Suh [37] showed that the additives effectively facilitated bubble formation by reducing the activation energy barrier to bubble nucleation at the interface between the additives and polymers, thereby promoting a high number of gas cells in the polymer foams. In the NR nanocomposite foams, the dispersed NC and CNF resulted in a large phase boundary or number of nucleation sites with low surface tension for bubble formation; thus, a great increase in cell density was obtained in the NC/NR and CNF/NR foams. Due to the better NC dispersion, a larger phase boundary was obtained in the NC/NR nanocomposite, which was confirmed by TEM analysis, as shown in the following text. Therefore, NC was more effective in foam formation. Moreover, with the reinforcing effect of NC and CNF, the rubbery materials in the cell walls became stronger and might have restricted the cell growth and cell coalescence, causing a decrease in cell size in the NR nanocomposite foams [14,26,28,29].

The foaming properties (density, volume expansion ratio, and shrinkage after foaming) of the foamed NR and NR nanocomposites are tabulated in Table 4. The density decreased due to the volume expansion following bubble formation in the NR matrix after foaming. As suggested from the smaller cell size and larger cell density in the NC/NR foam, as shown in Table 3, the density after foaming of the NC/NR foam was lower and the volume expansion ratio was larger than that of the CNF/NR foam. In order to determine the dimensional stability after the foaming of the NR and nanocomposite foams, the changes in the volume of the NR foam before foaming and after the stabilization were measured and recorded in terms of shrinkage. The obtained shrinkage is also tabulated in Table 4.

The shrinkage of the NR foam decreased after incorporating NC and CNF into the NR, indicating that the addition of nanofillers could significantly reduce the shrinkage of the NR foams. Since the cell wall was not sufficiently strong to support the cell structure in the NR foam, a substantial amount of shrinkage occurred after foaming to reduce the volume expansion. On the other hand, the addition of nanofillers could greatly decrease the foam shrinkage. The suppression of the shrinkage by NC and CNF might be attributed to their reinforcement of the cell walls. Owing to the greater suppression of shrinkage in the NC/NR foam, the density was lower and the volume expansion ratio was higher in the NC/NR foam than in the CNF/NR foam. For the NR nanocomposite foams, the greater suppression of shrinkage in the NC/NR foam was due to the greater reinforcement of the NR foam by NC dispersion. The reason for the stronger reinforcing effect of the NC/NR foam than the CNF/NR foam will be discussed later.

### 3.2. Dispersion of Nanofiller in NR Foams

It has been suggested that the dispersion of nanofillers inside the cell walls of polymer foams largely contributes to an improvement in the mechanical properties [29,32]. To clarify the dispersion of nanofillers in the NR nanocomposites after foaming, TEM observations were carried out before and after foaming.

**Figure 3:**
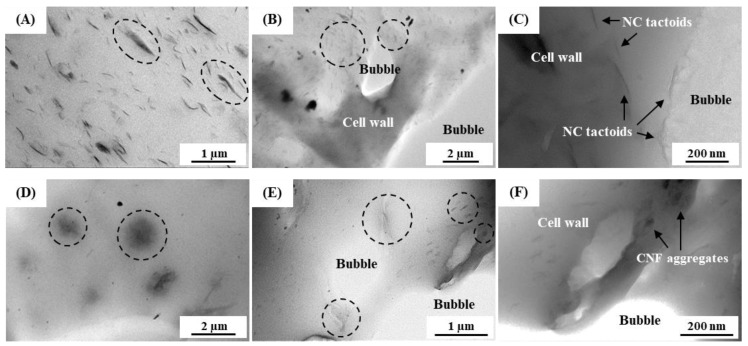
TEM images of (**A**) solid NC/NR (×10,000), (**B**) foamed NC/NR (×4000), (**C**) foamed NC/NR (×50,000), (**D**) solid CNF/NR (×5000), (**E**) foamed CNF/NR (×12,000), (**F**) foamed CNF/NR (×25,000).

Figure 3 shows the TEM images for cross-sections of the solid and foamed NR nanocomposites with 5 phr NC and CNF before and after foaming. The solid NC/NR showed dispersed NC tactoids (as indicated by the circles) in the NR matrix (Figure 3A). The thickness of the dispersed NC tactoids in the solid NC/NR measured by Image J software was about 23–113 nm. On the other hand, the CNFs were aggregated as large domains (in the circles) with dimensions ranging between 1 and 3 µm in the solid CNF/NR (Figure 3D).

An interesting result here was that the sizes of the NC tactoids and aggregated CNFs in the NR nanocomposite foams (Figure 3B,C,E,F) were much smaller than those observed in the unfoamed solid samples (Figure 3A,D). The dimensions of the NC tactoids and aggregated CNF particles in the foamed NR nanocomposites were about 3–34 nm in the NC/NR foam and 89–455 nm in the CNF/NR foam. These results indicate that the foaming of the NR nanocomposites caused a finer dispersion of nanofillers. These observations may be explained by the fact that a portion of gaseous N_2_ and CO_2_ molecules from the dissociation of ADC during the foaming process penetrated the interlayers of the NC and the interstitial spaces of the CNF aggregates, and these absorbed N_2_ and CO_2_ gases expanded upon the release of pressure from the foaming of the NR nanocomposites. The expansion of the blowing gases broke up the NC tactoids and aggregated CNFs, leading to smaller particles in both cases [32,38,39].

Upon foaming, the NC tactoids and CNF aggregates were located in the bulk region of the cell wall in the NR nanocomposite foams (Figure 3B,E), and some of them were aligned near the cell walls (Figure 3C,F). A similar result was also observed for oriented clay platelets at the cell wall in polyurethane (TPU) foams by Wang et al. [32]. The oriented NC at the cell wall was attributed to stresses from the equiaxial elongational flow generated by bubble expansion, and it significantly enhanced the elastic behavior of the TPU foams. In the case of the NR nanocomposite foams developed in this study, the alignment of nanofillers in the cell walls efficiently reinforced the NR foams by increasing their elastic properties; therefore, the gas cells in the NR nanocomposite foams were seen to be smaller and more spherical than those in the NR foam, as mentioned before.

### 3.3. Stress–Strain Behavior

**Figure 4:**
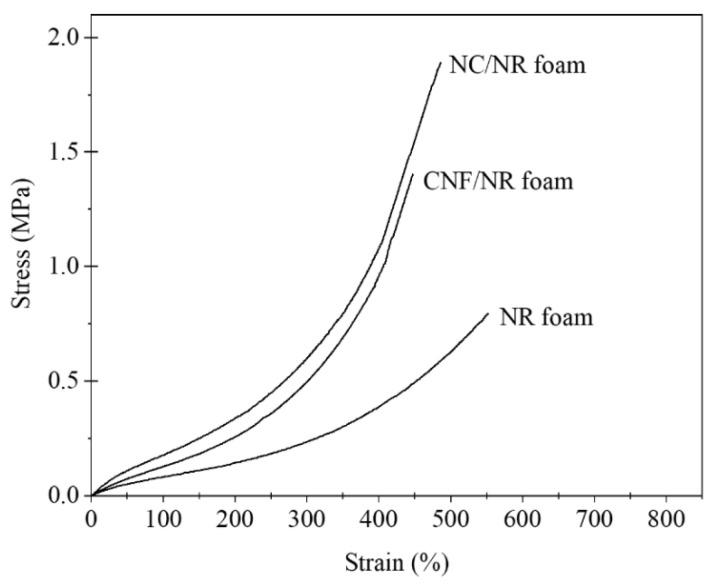
Stress–strain curves of NR, NC/NR, and CNF/NR foams.

Figure 4 shows representative stress–strain curves for the NR, NC/NR, and CNF/NR foams. The tensile stresses of the NR foam gradually increased with an increasing applied strain and sharply increased at a high strain of about 370%, as observed in the unfoamed solid NR included in our previous paper [34]. Obviously, the stress–strain curves of the NC/NR and CNF/NR foams filled with 5 phr nanofillers were similar to that of the NR foam, i.e., the tensile stresses remained low at a small strain and rapidly increased at high strains of about 150%.

The tensile stresses of the NR nanocomposite foams were substantially higher than those of the NR foam, i.e., the tensile stress were in the order of NC/NR foam > CNF/NR foam > NR foam.

**Figure 5:**
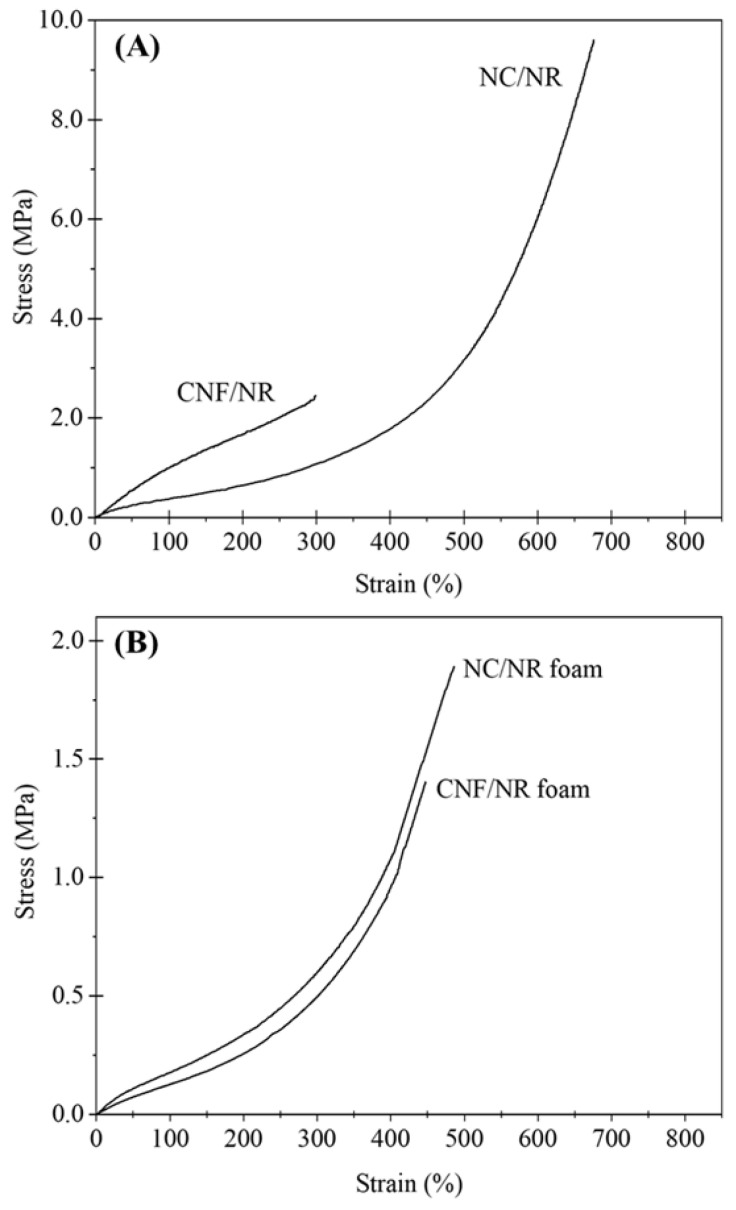
Comparison of stress–strain curves between (**A**) solid NR nanocomposites and (**B**) foamed NR nanocomposites.

Figure 5 shows the stress–strain behavior of the NR nanocomposites before and after foaming. Generally, the strength of the nanocomposite foams was lower than that of the corresponding solid samples due to the presence of bubbles, i.e., the presence of bubbles usually reduced the strength of the foam to 1/3 of the strength of the solid sample or lower. It is interesting to see that the stress–strain behavior of the nanocomposite foam (CNF/NR foam) was different to that of the unfoamed solid nanocomposite (CNF/NR).

The solid CNF/NR exhibited higher tensile stress but lower strain at break than the solid NC/NR, and no upturn in tensile stress was observed (Figure 5A). In contrast, the nanocomposite foams exhibited similar stress–strain behavior, and the tensile stress of the CNF/NR foam was lower than that of the NC/NR foam (Figure 5B). An interesting result here was that a stress upturn was observed in the CNF/NR foam, though no stress upturn was observed in the solid CNF/NR. The difference in the stress–strain behavior of the CNF/NR foam and solid CNF/NR might be attributed to the different dispersion states of the nanofillers, as shown in Figure 3D–F, i.e., large aggregated CNFs with a micron-sized scale of 1–3 µm existed in the solid CNF/NR, whereas much smaller aggregated CNFs with a size of 89–455 nm were dispersed in the CNF/NR foam. The large CNF aggregates in the solid CNF/NR induced highly stressed regions at the CNF/NR interface, leading to a great increase in tensile stress but inducing fracture at a low strain of about 300% [34,40]. On the other hand, the small CNF aggregates dispersed in the cell walls of the CNF/NR foam permitted large deformation without fracture [41], which increased the breaking strain of the CNF/NR foam when compared with the solid CNF/NR. As a consequence, strain-induced crystallization and a stress upturn were achieved in the CNF/NR foam.

The tensile strength increased by approximately 139% and 62% for the NC/NR and CNF/NR foams, respectively. In contrast, the strain at break of the NR foams (528%) decreased after incorporating NC (489%) and CNF (452%) due to the inclusion of stiff nanofillers. The decrease was small, indicating that the tensile strength of the NR foam was enhanced without sacrificing the strain by incorporating NC and CNF. As we have clearly demonstrated, the incorporation of nanofillers into the NR foams resulted in small spherical cells and a more homogeneous cell size distribution. Therefore, it is reasonable to conclude that the finer cellular structure and reinforcing effects of nanofillers increased the strain-induced crystallization ability of the NR in the cell walls of the foamed NC/NR and CNF/NR. These two main factors were responsible for the great improvement in tensile strength in the NR nanocomposite foams. For the NR nanocomposite foams, the NCs appeared to provide a better foam morphology and finer filler dispersion in the cell wall, resulting in a greater enhancement in the tensile modulus and strength for the NR foams when compared with the CNF.

To confirm the effects of the cellular structure and nanofillers on enhancing the crystallization in the NR nanocomposite foams during tensile deformation and compare their strain-induced crystallization, WAXD analysis was performed, and the results are discussed in the next section.

### 3.4. Strain-Induced Crystallization

**Figure 6:**
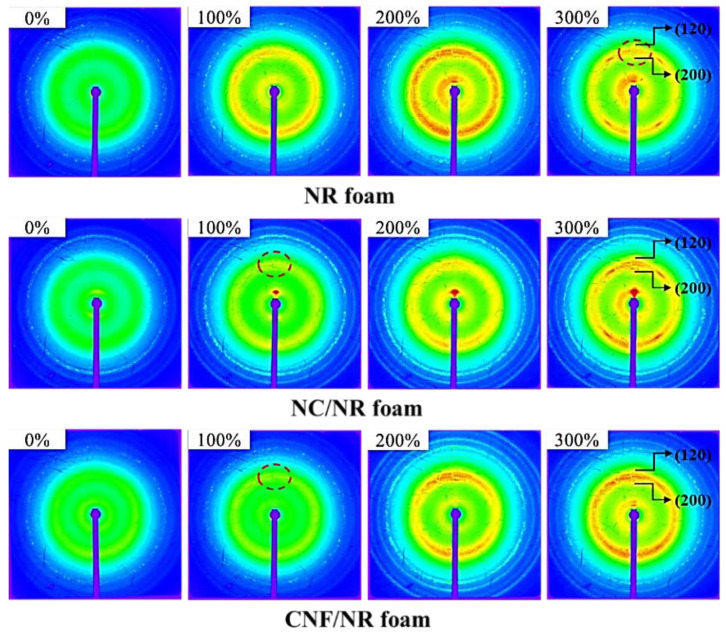
Representative 2D WAXD images of NR, NC/NR, and CNF/NR foams during stretching at various strains.

Figure 6 shows two-dimensional (2D) WAXD images of the NC/NR and CNF/NR foams during stretching at various strains. The 2D pattern of the pure NR is also included for comparison.

The 2D WAXD patterns of all foamed samples showed no reflection spot at 0% strain, indicating that crystallites did not exist, though an equiaxial elongational flow was generated by bubble expansion during the foaming. Upon stretching, the NR chains started to deform and aligned in the stretching direction. When the NR chains were fully stretched, highly oriented crystallite reflection spots were observed in the NR and NR nanocomposite foams. For the NR foam, the reflection spots assigned to the (200) and (120) planes were detected at a strain of about 300%, as illustrated by the dashed circles. The reflection spots were attributed to the strain-induced crystallization usually observed in solid NR during stretching. Thus, strain-induced crystallization occurred in the cell structure of the NR foam. Highly oriented reflection spots appeared at a much lower strain of about 100% in the NC/NR and CNF/NR foams. This suggested that the strain-induced crystallization occurred faster in the NC/NR and CNF/NR foams than in the pure NR foam. Thus, the strain-induced crystallization of the NR foam was accelerated by incorporating nanofillers. The acceleration of the strain-induced crystallization in the NR nanocomposite foams might be attributed to the early alignment of NR chains inside the cell wall of the NR nanocomposite foams due to the fine dispersion of nanofillers.

**Figure 7:**
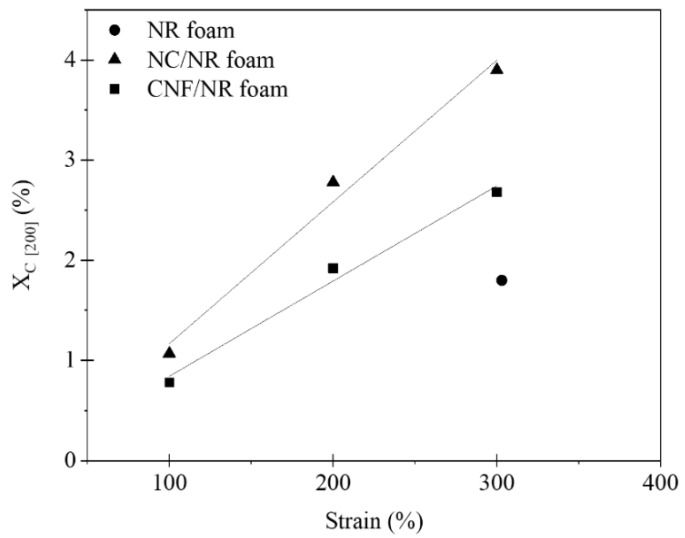
Variation in crystallinity (*X_c_*) as a function of applied strain for NC/NR and CNF/NR foams in comparison with the crystallinity of the NR foam.

Figure 7 displays the degree of crystallinity (*X_c_*) of the NC/NR and CNF/NR foams as a function of the applied strain, in the range of 100–300%. The crystallinity of the NR foam is also included in Figure 7 for comparison. Apparently, the crystallinity of the nanocomposite foams increased with an increasing strain due to strain-induced crystallization. The onset of strain-induced crystallization in the NR foam was observed at a high strain of about 300%, while that of the nanocomposite foams was observed at a much lower strain of about 100%.

In the unfilled pure NR, it was shown that the strain-induced crystallization during stretching was initiated from the crosslinked network of NR, as discussed in our previous work [34]. Therefore, the crystallization during stretching in the pure NR foam was attributed to the crosslinking points in the NR foam. The strain-induced crystallization was accelerated in the nanocomposite foams. This could be explained by the rotation of well-dispersed NC particles in the cell wall of the NC/NR foam and the immobilizing effect of NR chains at the surface of the small CNF aggregates dispersed in the cell wall of the CNF/NR foam [34]. It is interesting to note that the NR nanocomposite foams showed a lower onset strain for crystallization (about 100%) than the solid NR nanocomposites (about 150%), while the onset strain for crystallization was not changed in the pure NR, i.e., the onset strains for crystallization in both the solid NR and NR foam were about 300%. This was probably because some of the NR molecules attached to the surfaces of the NC and CNF located at the boundary between the cell walls and bubbles were oriented (Figure 3B,C,E,F) and crystallized upon stretching. As shown in Figure 4, a sharp increase in the stress occurred around the same strain at which the strain-induced crystallization started to occur.

Thus, the results obtained from the WAXD measurements also emphasized the contribution of strain-induced crystallization to the improvements in the tensile properties of the NR nanocomposite foams. It was seen that the crystallinity of the NC/NR foam was higher than that of the CNF/NR foam. Since the NC/NR foam had a smaller cell size and higher cell density than the CNF/NR foam (Figure 2), the well-dispersed NC in the NR matrix was more effective in accelerating the strain-induced crystallization of the NC/NR foam when compared with the CNF/NR foam. The improved cellular structure also caused a significant increase in the cell surface area for molecular orientation [42]. Therefore, the increase in crystallinity was more pronounced when the NR foam was reinforced with NC. The results based on morphological observations, stress–strain behavior, and WAXD measurements showed that the acceleration of strain-induced crystallization related to a fine cellular structure and nanofiller addition resulted in an increase in the strain-induced crystallization of the NR foams, which caused an improvement in the tensile properties of the nanocomposite foams. That is to say, for the nanocomposite foams, a sharp increase in the stress occurred at a lower strain and the stress was higher compared the NR foam, as shown in Figure 4.

### 3.5. Model for Strain-Induced Crystallization of CNF/NR Foams

**Figure 8:**
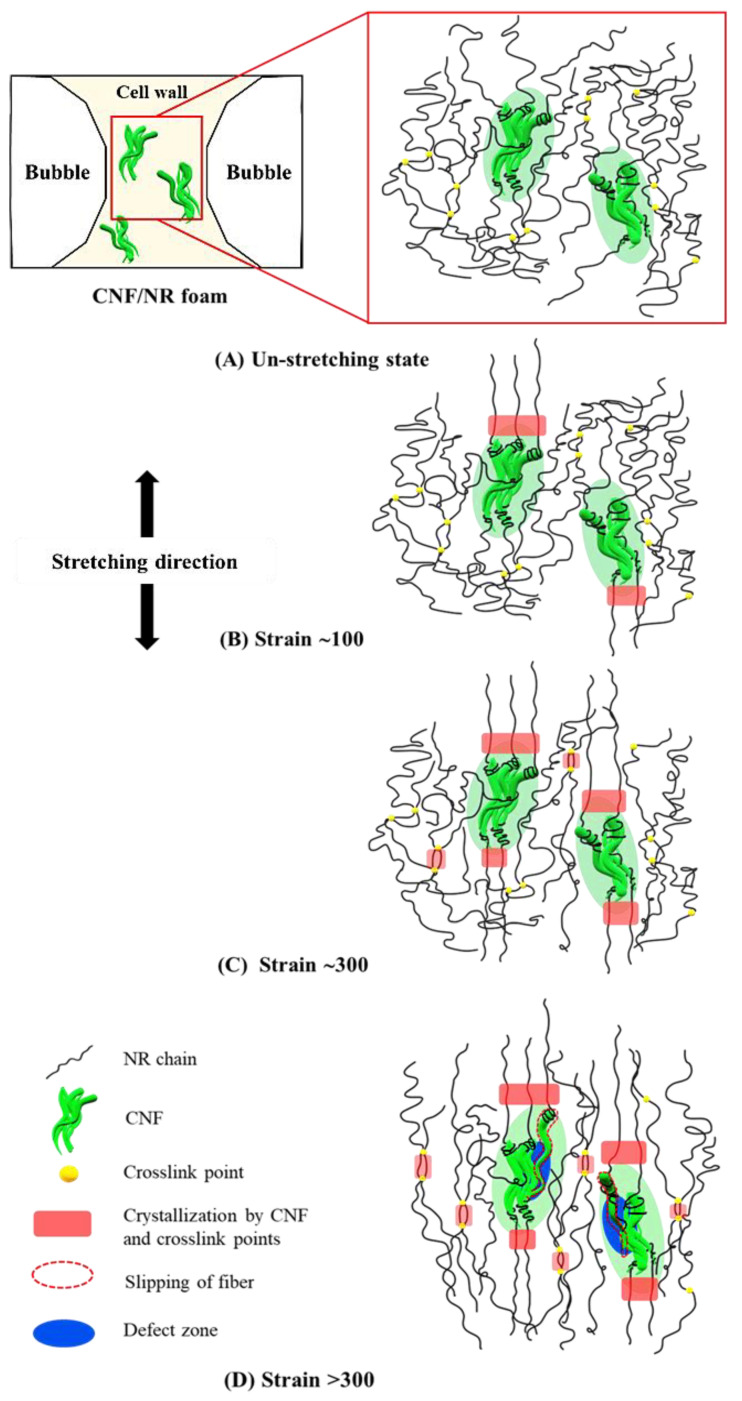
Proposed model for strain-induced crystallization of CNF/NR nanocomposite foam under (**A**) no stretching, (**B**) about 100% strain, (**C**) about 300% strain, and (**D**) over 300% strain.

The solid NC/NR and corresponding NC/NR foam showed similarities in their stress–strain curves and high upturns in stress during tensile stretching due to the orientation of well-dispersed NC [34,43,44]. On the other hand, the stress–strain characteristics of the solid CNF/NR and foamed CNF/NR were very distinguishable. The solid CNF/NR provided a significant increase in tensile stress from a low strain, and it failed at a low strain with no upturn in stress. Surprisingly, the CNF/NR foam showed an upturn in stress and a high strain at break. These results suggest that the reinforcing effects were different. The reinforcement mechanism of the solid CNF/NR (5 phr CNF) was believed to result from the reinforcing effect of micron-sized CNF aggregates [34,40]. It is worth noting that the CNF aggregates became much smaller through the foaming process. A model depicting the proposed reinforcing effect of finely dispersed CNF in the NR nanocomposite foam is shown in Figure 8.

In the unstretched state, small aggregated CNFs were locally dispersed in the cell wall in the CNF/NR foam, and it was assumed that they contained immobilized NR chains due to the insertion of NR chains in the CNF aggregates [34] (Figure 8A). When stretching to 100% strain, the immobilized NR chains at the surfaces of the aggregated CNFs in the cell wall were initially oriented and crystallized, as revealed by the 2D WAXD image shown in Figure 6 (Figure 8B).

Upon further stretching, the crystallites of the CNF/NR foam increased due to the strain-induced crystallization of immobilized NR chains, leading to stronger intensities of crystalline reflection. As the tensile deformation reached a strain of 300%, the short NR chains between the crosslinked point could be orientated and formed crystallites [34,43,44] in addition to the strain-induced crystallization by CNF dispersion, leading to a high degree of crystallinity in the CNF/NR foam (Figure 8C). This caused a significant enhancement in stress at 300% strain for the CNF/NR nanocomposite foam (an increase of about 129%) when compared with the neat NR foam. Upon tensile stretching to a strain greater than 300%, the entangled CNFs in the nanofiber aggregates began to slip or slide against each other, which gave rise to the formation of defects in the cell wall of the CNF/NR foam. Under these circumstances, the cell wall ruptured, leading to the failure of the CNF/NR foam at higher deformation levels (>300%) (Figure 8D).

### 3.6. Compression Set of NR and NR Nanocomposite Foams

**Figure 9:**
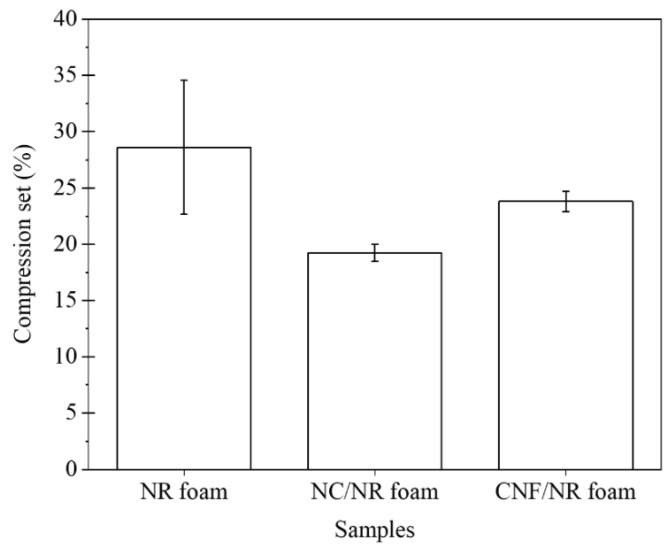
Compression sets of NR, NC/NR, and CNF/NR foams.

The compression sets of the NR and NR nanocomposite foams are compared and presented in Figure 9. The compression set is a measure of the residual deformation in an elastomer after the removal of a given compressive force. This measurement reveals the elastic recovery and resilience of rubbery materials [4]. Basically, elastomers with a high compression set do not return to their original thickness, while those with low compression set values recover most of their original thickness when the force is removed. The compression set of the NR foam substantially decreased after the incorporation of nanofillers. The compression set values of the NC/NR and CNF/NR foams were 28% and 17% lower than that of the NR foam, respectively, indicating the improved elastic recovery of the NR foams in the presence of nanofillers. These improvements were attributed to the dispersion of nanofillers in the cell walls, which could effectively enhance the elastic properties and resistance to permanent deformation during compressive loading. Furthermore, the smaller cell size and narrower cell size distribution observed in the NR foams when incorporating nanofillers, particularly NC, suppressed the permanent set due to the more uniform stressed state in the samples.

Based on the above results, it is suggested that the addition of nanofillers could substantially improve the compression set of foamed NR, which is desirable for dynamic seals and gaskets, where elastic recovery is important.

## 4. Conclusions

NR nanocomposites were prepared by adding two kinds of nanofillers, NC and CNF, at an amount of 5 phr and later foamed by a compression-molding process using ADC as the foaming agent.

The addition of nanofillers could remarkably improve the cell structure of the NR foams and suppress shrinkage after foaming. Smaller cells with a narrower distribution were attained in the NC/NR foam, and the expansion ratio was larger due to the suppression of the shrinkage when compared with the NR and CNF/NR foams, e.g., the average cell size of the NC/NR, NR, and CNF/NR foams were 29, 61, and 42 µm, respectively. The cell density of the NC/NR, NR, and CNF/NR foams were 2.4, 0.09, and 0.37 × 10^7^ cells/cm^3^, respectively.

The foaming of the nanocomposites resulted in a reduction in the aggregate size of the nanofillers and the fairly homogeneous dispersion of the nanofillers in the cell walls. Strain-induced crystallization was found to occur in the NR foam during stretching, and it was accelerated in the NR nanocomposite foams due to the rotation of the well-dispersed NC and the immobilizing effect of the aggregated CNFs. Owing to the accelerated crystallization, the tensile strength of the NR foam was enhanced by adding nanofillers, i.e., the stress at break of the NC/NR, NR, and CNF/NR foams was 1.9, 0.78, and 1.4 MPa, respectively.

The addition of nanofillers also decreased the compression set of the NR foams due to the increment in elasticity and the improvement of the cellular structure in the NR foams, i.e., the compression sets of the NC/NR, NR, and CNF/NR foams were 20, 28, and 24%, respectively. The good elastic recovery of the NR nanocomposite foams suggested that these materials could be beneficial for dynamic sealing and gasketing applications.

## Figures and Tables

**Table 1:** List of rubber, fillers, and additives for NR and NR nanocomposite foams.

Ingredient	Grade	Supplier
NR	HA	Yala Latex Co., Ltd., Yala, Thailand
NC (Na-MMT)	Kunipia-F^®^	Kunimine Industries Co., Ltd., Tokyo, Japan
CNF	Nanoforest-S	Chuetsu Pulp and Paper Co., Ltd. Tokyo, Japan
ZnO	White seal	Univentures Public Co. Ltd., Bangkok, Thailand
Strearic acid	Strearic acid	Imperial Chemical Co. Ltd., Bangkok, Thailand
Paraffinic oil	White oil grade A, no. 15	China Petrochemical International Co., Ltd., Shanghai, China
TMQ	NAUGARD^®^ Q	Addivant^TM^, Latina, Italy
ADC	AC3000F	Innovation Co. Ltd., Bangkok, Thailand
DCP	GP grade	Wuzhou International Co., Ltd., Shenzhen, China

**Table 2:** Formulation of NR and NR nanocomposite foams.

Ingredients	Parts per Hundred Rubber (phr)		
	NR	NC/NR	CNF/NR
NR	100	100	100
NC (Na-MMT)	-	5	-
CNF	-	-	5
ZnO	3	3	3
Strearic acid	1	1	1
Paraffinic oil	20	20	20
TMQ	2	2	2
ADC	10	10	10
DCP	1	1	1

**Table 3:** Effect of NC and CNF addition on the cell properties of the NR foams.

Sample	Cell Properties		
	Cell Size Distribution (µm)	Average Cell Size (µm)	3D Cell Density (cells/cm^3^)
NR foam	15–153	60.85 ± 24.38	8.99 × 10^5^ ± 0.27 × 10^5^
NC/NR foam	4–87	28.86 ± 12.02	2.44 × 10^7^ ± 0.35 × 10^7^
CNF/NR foam	11–108	41.79 ± 16.83	3.68 × 10^6^ ± 0.23 × 10^6^

**Table 4:** Effect of NC and CNF addition on the foam properties of NR foams.

Samples	Foam Properties			
	Density (g/cm^3^)		Volume Expansion Ratio	Shrinkage (%)
	Before Foaming	After Foaming		
NR foam	0.913 ± 0.002	0.501 ± 0.013	1.823 ± 0.048	58.87 ± 3.83
NC/NR foam	0.943 ± 0.003	0.356 ± 0.023	2.660 ± 0.164	18.10 ± 6.65
CNF/NR foam	0.931 ± 0.007	0.393 ± 0.034	2.383 ± 0.203	35.89 ± 5.21

## Data Availability

The data presented in this study are available on request from the corresponding author.
